# A 6-month-old girl with mesenteric teratoma: A Case report and literature review

**DOI:** 10.3389/fped.2023.1131190

**Published:** 2023-03-15

**Authors:** Yuyan Jin, Yanan Zhang, Yongwei Chen, Jinshi Huang

**Affiliations:** Beijing Children’s Hospital, Capital Medical University, National Center for Children’s Health, Beijing, China

**Keywords:** teratoma, mesentery, infant, surgery, case report

## Abstract

An otherwise healthy 4-month-old girl presented to the community health service center because her abdomen was distended. Over the next 2 months, the girl's abdomen gradually became more distended. Her examination was notable for abdominal distention with a large, mobile, non-tender abdominal mass. Abdominal ultrasound images and subsequently obtained CT images showed a large, circumscribed cystic and solid mass. This led to the presumptive diagnosis of teratoma of the mesentery. The mass was completely resected during a laparotomy. The pathology, along with the surgical findings and imaging, led to the final diagnosis.

## Introduction

Teratomas are common tumors in infancy, and they display a mixture of tissues from at least two of the three germ layers. The most common sites are the midline and gonads of the body, such as the sacrocaudal area (57%), gonadal area (29%), mediastinum (7%), and retroperitoneum (4%) (1). Mesenteric teratomas are very rare, occurring in approximately 1/1,000,000 live births (2). Only a few cases have been published and described in contemporary literature (3–13). While a mesenteric cyst is a common mass of mesenteric origin and has a morbidity rate of 1 in every 20,000 pediatric hospital admissions, the diagnosis of mesenteric teratoma can be easily missed at the initial stage. (14). An asymptomatically increasing abdominal girth or a palpable mass is usually accompanied by other symptoms such as vomiting and constipation (7). Here, we describe a case of a patient with mesenteric teratoma.

## Case presentation

A 6-month-old girl was admitted to our hospital because of an abdominal mass. Approximately 2 months previously, the patient's parents had noticed that her abdomen had gradually become distended, but there were no other complaints. She was initially taken to the community health service center. On examination at the health service center, the patient appeared to be in normal condition. The patient's weight was 6.0 kg, the abdomen was mildly distended but soft, and there were normal bowel sounds and no apparent tenderness or mass on palpation. The examination concluded without any further diagnosis or prescribed treatment.

The patient had been born by cesarean section because of preeclampsia after 38 weeks of gestation. She weighed 3.2 kg at birth and was fed breast milk. Supplements were added, and mixed feeding had begun when she was 5 months old. The patient's childhood immunizations were up to date. She had no known medical conditions, had not received any additional medications, and had no known allergies. She lived with her parents and grandparents. There was no family history of cancer.

On examination at the hospital, the patient was placid. Her temperature was 36.6°C, pulse was 128 beats per minute, respiratory rate was 30 breaths per minute, and oxygen saturation was 98% while she was breathing ambient air. The patient's weight was 8.5 kg (89th percentile), her height was 69 cm (93rd percentile), and her head circumference was 42.0 cm (44th percentile). The abdomen was soft and distended with a circumference that measured 50 cm. On palpation, there was a large mobile non-tender abdominal mass that measured approximately 19 cm × 15 cm. There were no other abnormal physical findings. Blood levels of glucose, sodium, potassium, chloride, creatinine, total protein, albumin, globulin, and lactate dehydrogenase were normal, as were the results of coagulation and liver function tests. The other test results are given in Table 1.

**Table 1:** Laboratory data.

Variable	Reference range, age-adjusted^a^	On admission, this hospital
Hematocrit (%)	32–45	37.2
Hemoglobin (g/dL)	103–138	121
Red-cell count (10^12^/L)	4.1–5.5	4.97
White-cell count (10^9^/L)	5.0–14.2	9.24
Differential count (%)		
Neutrophils	9–53	17.5
Lymphocytes	37–82	75.1
Monocytes	2–14	4.3
Eosinophils	0.8–11	2.9
Basophils	0.0–1.0	0.2
Platelet count (10^9^/L)	172–601	415
Sodium (mmol/L)	134–143	137.2
Potassium (mmol/L)	4.2–5.9	5.48
Chloride (mmol/L)	98–110	105.7
Urea (mmol/L)	1.1–5.9	1.76
Creatinine (μmol/L)	13–33	15.7
C-reactive protein (mg/L)	0.00–9.00	<8
Alpha-fetoprotein (AFP) (ng/mL)		32.64
Luteinizing hormone (IU/L)		0.000
Serum follicle-stimulating hormone (IU/L)		3.17
Testosterone (ng/dL)		<7.00
Estradiol (pg/mL)		24.16
Prolactin (ng/mL)		6.55
Serum progesterone (ng/mL)		0.26
Sex hormone–binding globulin (nmol/L)		>180.00
Neuron-specific enolase (ng/mL)		20

^a^ Reference values are affected by many variables, including the patient population and the laboratory methods used. The ranges used at Beijing Children's Hospital are adjusted for patients aged 28 days to 18 years. They may therefore not be appropriate for all patients.

An ultrasonography of the abdomen confirmed the presence of a mass in the patient's enterocoelia that measured 15.9 cm × 7.9 cm × 17.1 cm. The mass presented as a mixture of echoes and was well circumscribed; it mainly consisted of fat echo (multiple linear hyperechoic areas inside isoechoic areas) with many cysts of various sizes (the largest one measured 6.2 cm × 3.6 cm × 4.4 cm) and several hyperechoic areas with acoustic shadowing. The large retroperitoneal vessels were squeezed and shifted to the back of the patient, but they were not wrapped, and she did not exhibit any other abnormalities relating to any other organs.

The ultrasonography indicated that the mass in the abdomen of the patient was significant in size and would be difficult to remove. Therefore, we needed to perform a computer tomography (CT) scan to clarify the origin of the mass and identify the surrounding vessels.

**Figure 1:**
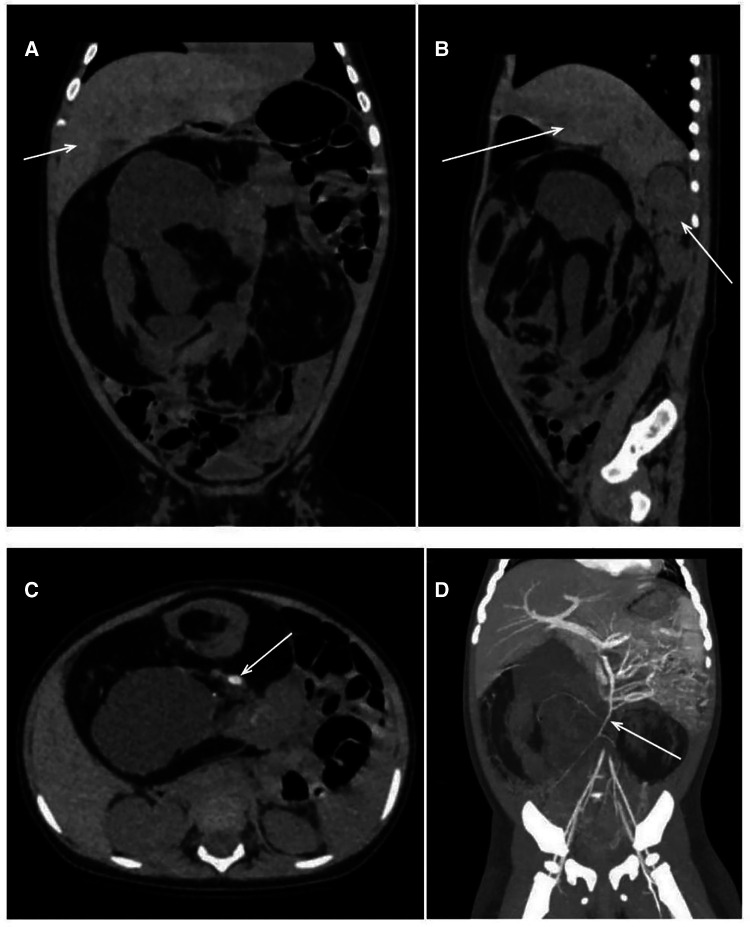
CT obtained at this hospital. CT images (**A**, **B**), obtained before the administration of intravenous contrast material, show a multilobulated cystic mass compressing the liver and right kidney (arrows). The CT image (**C**) shows a mixed density of mass, including a calcified shadow (arrow). The CT image obtained after the injection (**D**) confirms that the structure was supplied by the superior mesenteric artery (arrow). CT, computed tomography.

The CT scan depicted a huge space-occupying lesion that appeared to be of mixed density, and the mass reached both the liver's lower edge and the pelvic cavity (Figures 1A,B). The lesion had a maximum diameter of 15.0 cm × 13.5 cm × 9.5 cm and showed an evident mass effect containing various densities, such as fluid (CT value −128 HU), fat (CT value 12 HU), soft tissue (CT value 38 HU), and calcification/ossification shadows (CT value 1,220 HU) (Figure 1C). On contrast-enhanced CT, the soft tissue density was found to be enhanced with a CT value of 95 HU, and there was a vascular shadow that could be seen in the lesion. The superior mesenteric artery branches extended inward, while the other large abdominal vessels and branches were compressed (Figure 1D). As the lesion occupied the void of the abdominal cavity, the intra-abdominal pressure increased. Therefore, the internal organs (the intestines and kidneys) that are not entirely fixed tended to be displaced upward, while the relatively fixed organ (the liver) was flattened. On the contrast-enhanced CT scan, the abdominal parenchyma organs were found to be enhanced uniformly, and no lumps or defects were noted.

## Differential diagnosis

Abdominal masses found in children are associated with a broad differential diagnosis because of the various underlying causes; they may originate from one of several possible structures (hepatobiliary system/kidney/pancreas/spleen/adrenal gland/ovarian/mesentery/bowel) (15, 16), and the mass may be found incidentally or with obvious symptoms (abdominal pain/bowel obstruction/fever/early satiety) (17). This 6-month-old patient presented only with a movable abdominal mass.

Usually, the common abdominal masses in this age group are neuroblastoma, mesenteric lymphangioma, retroperitoneal teratoma, and ovarian teratoma. Findings on the initial ultrasonography examination suggested that the mass was well circumscribed and had mixed echogenicity (containing fat echo, plentiful cysts, and hyper echoes). The diagnosis of neuroblastoma was excluded because solid masses would have been present anywhere throughout the sympathetic nervous system, with the adrenal gland being the most common primary site. Using CT scans, the diagnosis was clarified because both cystic and solid components could be seen, including fat and calcified/ossific shadows. The diagnosis of mesenteric lymphangiomas was excluded because such tumors are cystic masses without calcification. The diagnosis of teratoma became evident. Therefore, the focus now turned to the origin of the teratoma. Although retroperitoneal teratomas are more common in children, a diagnosis of a retroperitoneal teratoma was excluded because the patient's ultrasonography (US) and CT indicated that the teratoma was located in the abdominal cavity. After the administration of intravenous contrast material, the supplying vessels were recognized as the branches of the superior mesenteric artery, which meant that the mass originated from the mesentery, and the diagnosis of an ovarian teratoma was excluded. Furthermore, the patient had normal ovaries and normal levels of sex hormones.

Our initial impression was that this patient had a large, polycystic mass that seemed to be a mesenteric teratoma. Because mature teratomas are much more common than immature teratomas in infants, mature mesenteric teratomas were first considered in this patient.

## Management

After an extensive discussion with her parents, a decision was made to proceed with a laparotomy. The patient had a preoperative diagnosis of a mesenteric teratoma that required complete resection.

**Figure 2:**
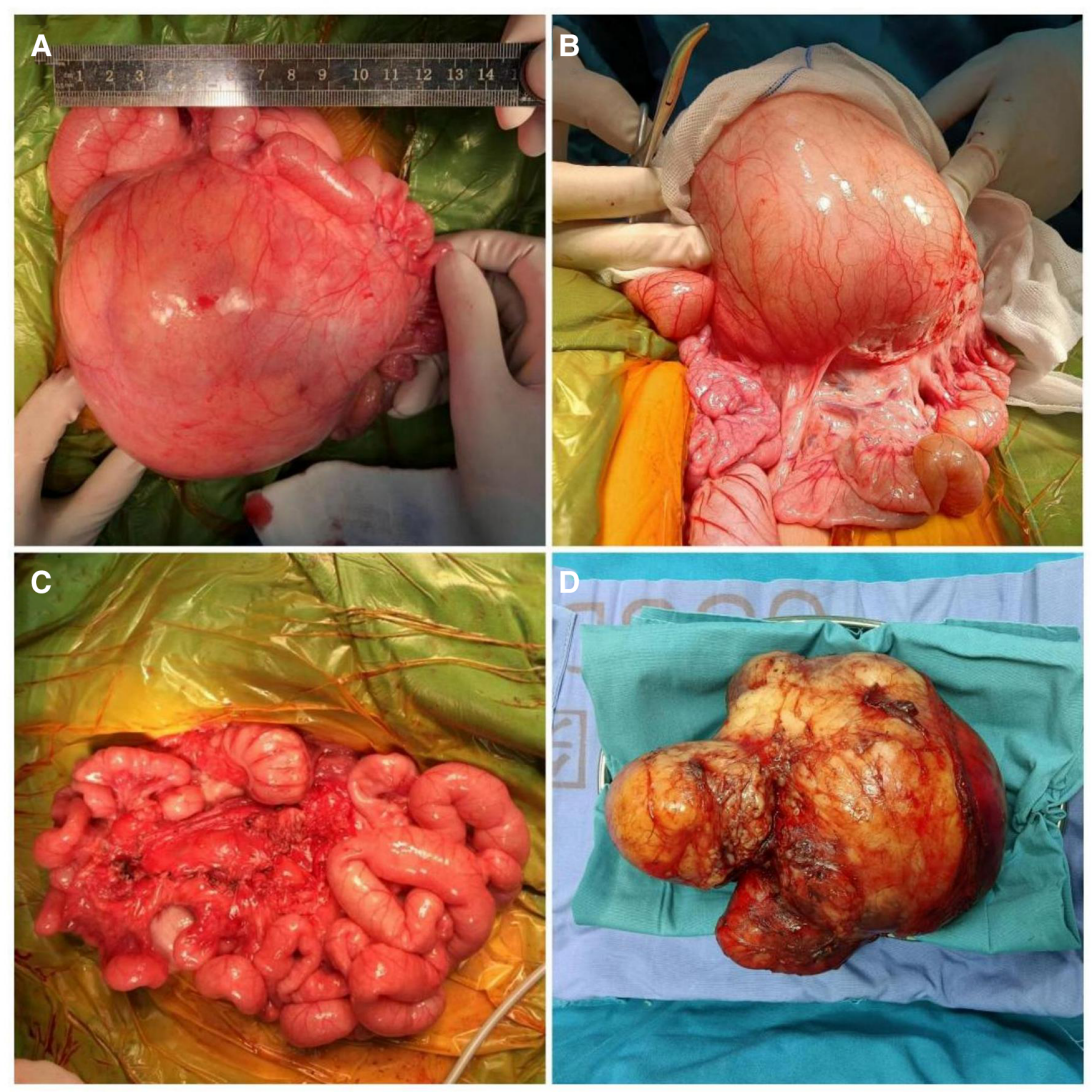
Pictures of a mass obtained during the operation. Pictures (**A**, **B**) show that the mass originated from the mesentery. Pictures (**C**, **D**) were taken after the mass was resected.

The abdomen was entered through a transverse upper abdominal incision, and a pineapple-sized multiple cystic mass was found (Figures 2A,B). The superior mesenteric artery branches not only wrapped around the mass but also gave rise to arteries that had entered the mass. The vessels supplying blood to the mass were ligated, and the vessels supplying blood to the intestine were separated. Then, the mass was resected intact at the radix of the mesentery (Figure 2C). We finally sutured the hiatus of the mesentery (Figure 2D). None of the intestines were removed. The patient was discharged home with an abdominal circumference of 40 cm and a weight of 7.8 kg (70.5th percentile) on postoperative day 7.

## Pathological diagnosis

**Figure 3:**
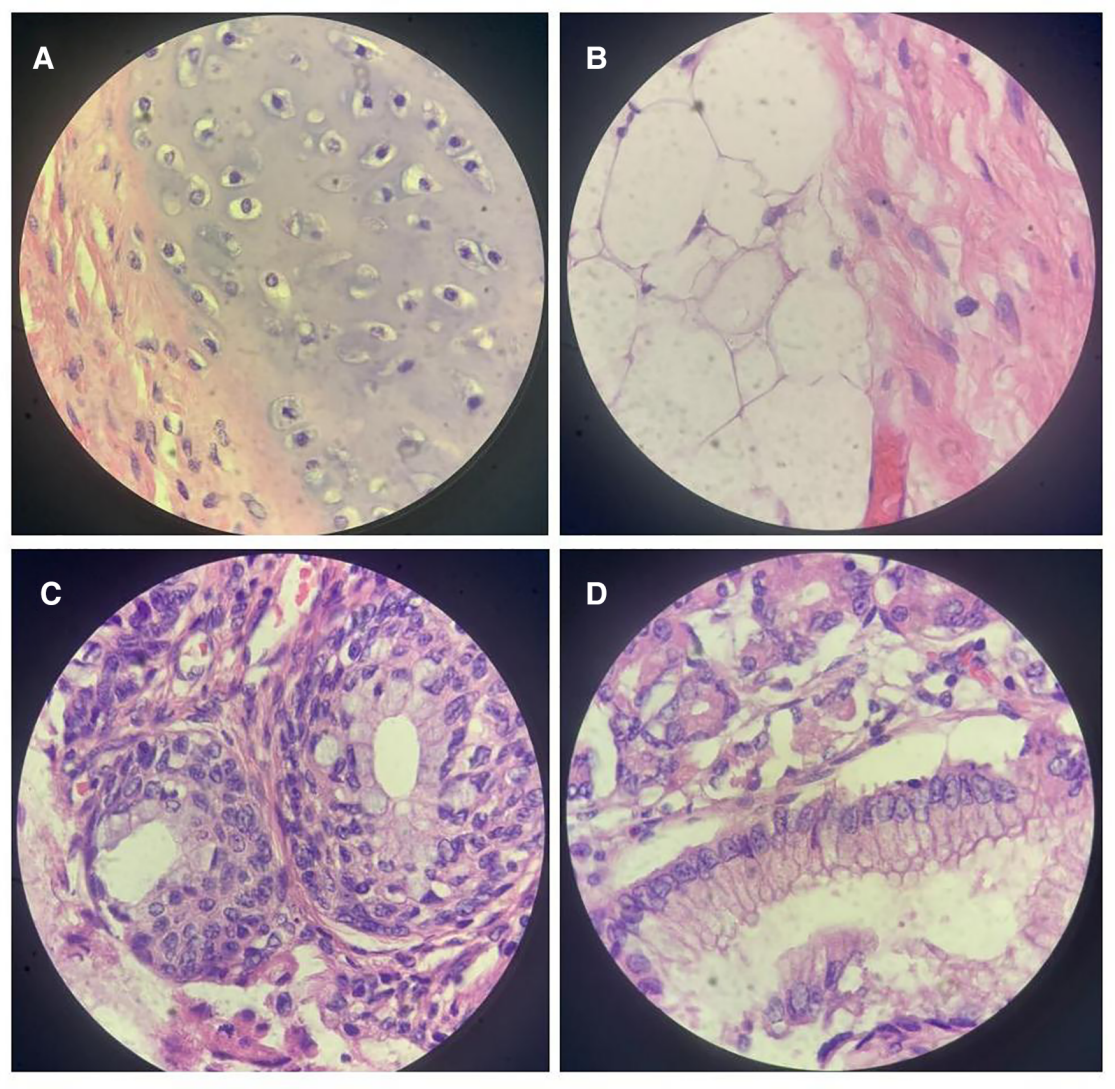
High-power view of the tumor. A microscopic examination revealed cartilaginous tissue (**A**), adipose tissue (**B**), glandular tissue (**C**), and epithelial tissue (**D**).

The pathological diagnosis was a (Celiac) solid and cystic mature teratoma (WHO 0). The size of the mass was 14.5 cm × 12 cm × 11 cm. Most parts of the surface were covered with capsules, the nature of the surface ranged between dark purple and sallow, and the mass was smooth. In profile, it was solid with cysts of 0.5–5.5 cm in diameter. The capsule contained a bright viscous liquid, and the remaining solid area was sallow and felt greasy to the touch. Cartilaginous, adipose, glandular, and epithelial tissues were observed (Figure 3).

## Follow-up

The patient recovered well with no subsequent complications and was 9.5 kg (88th percentile) in weight and 73 cm (88th percentile) in height with an abdomen circumference of 49 cm. An ultrasonography revealed that there was no stenosis in the initial segment of the superior mesenteric artery and that blood flow was not restricted. The alpha-fetoprotein (AFP) level dropped to 4.65 ng/mL.

## Final diagnosis

The final diagnosis was a mature teratoma of the mesentery (WHO 0).

## Discussion

Only 12 cases of mesenteric teratoma have been reported worldwide (Table 2). Except for one case that was a fetus, the rest of the cases were patients who ranged in age from 2 months to 9 years, and 8 of the 12 patients were female (58.3%). Half of the patients presented with an asymptomatic abdominal distention/mass, while the other half presented with accompanying symptoms such as vomiting (36.4%), constipation (27.3%), abdominal pain (18.2%), and diarrhea (9.1%). Most of the cases were mature mesenteric teratomas (66.7%), and only one case was multiple mesenteric teratomas.

**Table 2:** The 12 cases of mesenteric teratomas previously reported in children.

Case No.	References	Year	Age	Sex	Clinical symptoms	Pathological diagnosis	Imaging methods
1	Prieto et al. (3)	1989	5 years	F	Abdominal mass	Cystic teratoma	US
2	Chiba et al. (4)	1995	8 months	F	Diarrhea	Mature teratoma	US, CT
3	Marcolongo et al. (5)	1997	1 day	M	—	Immature teratoma	US, x-ray
4	Ratan et al. (6)	2002	2 years	M	Abdominal distension, constipation, and vomiting	Cystic mature teratoma	US, x-ray
5	Al-Arfaj et al. (7)	2003	5 months	F	Abdominal distension	Mature teratoma	CT
6			4 months	M	Vomiting and constipation	Mature teratoma	US
7	Okada et al. (8)	2006	9 years	F	Abdominal pain and vomiting	Multiple mesenteric teratomas	US, CT, MR
8	Rattan et al. (9)	2007	7 years	F	Abdominal pain, vomiting, and constipation	Immature teratoma	US, x-ray, CT
9	Singh et al. (10)	2013	5 years	F	Abdominal distension	Mature teratoma	US, CT, x-ray
10	Zhang et al. (11)	2015	15 months	F	Abdominal mass	Cystic mature teratoma	CT
11	Li et al. (12)	2021	2 months	M	Abdominal distention	Immature teratoma	CT
12	Ray et al. (13)	2022	4 months	M	Abdominal mass	Immature teratoma	CT

US, ultrasound; CT, computed tomography; MR, magnetic resonance.

Reviewing the history of this patient, we noted that she had asymptomatic abdominal distention when she was 4 months old. The patient's weight was 6 kg (29th percentile) at the first visit, and when compared with her birth weight of 3.2 kg (47th percentile), her weight gain was slower than that of infants of the same age. However, 2 months later, the patient's weight increased dramatically from 6 kg (29th percentile) to 8.5 kg (89th percentile), which meant that the weight gain rate significantly accelerated because of unknown factors. The patient's abdominal distention gradually became obvious at the same time, indicating that the mass was growing rapidly.

In previous cases, all patients underwent ultrasonography, and the other imaging examinations were CT, x—ray, and magnetic resonance (MR). However, only three of these patients had been provisionally diagnosed with mesenteric teratoma before surgery. Two patients had been provisionally diagnosed with an ovarian mass and one other as having a tubercular mass. In the remaining patients, there was not sufficient information to determine the origin of the teratoma. While the diagnosis of teratoma can be relatively easy to establish through imaging findings such as ultrasound and CT (the mass contains multiple components such as bone, fat, and fluid), the origin of an abdominal teratoma is more difficult to determine. A mass located on the alimentary tract or related organs strongly suggests the diagnosis of gastrointestinal teratoma (2). The larger the mass is, the more difficult it becomes to determine the source by imaging findings. Combined with the fact that the mass was movable during the physical examination and that the superior mesenteric artery had vascular branches extending into the tumor, the results from further use of the enhanced CT scan indicated that the provisional diagnosis before surgery was a mesenteric teratoma. This provisional diagnosis was confirmed through surgical findings.

The treatment for mesenteric teratoma is surgery, and complete resection of the tumor is warranted. As teratomas always adhere to the root of the mesentery rather than to the distal mesentery, the supplying vessels are relatively independent. Thus, the tumor can be resected without interrupting the continuity of the intestine.

## Conclusions

Mesenteric teratomas are very rare teratomas that usually present initially with asymptomatic abdominal distension or digestive tract symptoms, and abdominal ultrasound is the first choice of examination.

In infants, a circumscribed mass located on the alimentary tract or related organs with cystic and solid components, including fat, fluid, and calcified/ossific shadows, suggests mesenteric teratoma.

All mesenteric teratomas should be surgically resected, and enhanced CT should be performed before surgery to clarify the vascular course and ensure that blood flow to the intestines is maintained.

## Data Availability

The original contributions presented in the study are included in the article/Supplementary Material, and further inquiries can be directed to the corresponding author.

## References

[1] Grosfeld JL Billmire DF. Teratomas in infancy and childhood. Curr Prob Cancer. (1985) 9(9):1–53. 10.1016/S0147-0272(85)80031-3 2415302

[2] Bowen B Ros PR McCarthy MJ Olmsted WW Hjermstad BM. Gastrointestinal teratomas: CT and US appearance with pathologic correlation. Radiology. (1987) 162(2):431–3. 10.1148/radiology.162.2.3541031 3541031

[3] Prieto ML Casanova A Delgado J Zabalza R. Cystic teratoma of the mesentery. Pediatr Radiol. (1989) 19(6–7):439. 10.1007/BF02387647 2671899

[4] Chiba T Iwami D Kikuchi Y. Mesenteric teratoma in an 8-month-old girl. J Pediatr Surg. (1995) 30(1):120. 10.1016/0022-3468(95)90627-4 7722815

[5] Marcolongo A Divirgilio G Bettili G Camoglio FS Fasoli L Marradi P. Immature mesenteric teratoma in a male newborn infant: prenatal ultrasonographic diagnosis and surgical treatment. Prenatal Diag. (1997) 17(7):686–8. 10.1002/(SICI)1097-0223(199707)17:7<686::AID-PD686>3.0.CO;2-5 9249872

[6] Ratan SK Ratan J Kalra R. Large benign cystic teratoma of the mesosigmoid causing intestinal obstruction: report of a case. Surg Today. (2002) 32(10):922–4. 10.1007/s005950200183 12376796

[7] Al-Arfaj AA El-Shawarby MA Al-Mulhim FA Lardhi AA. Mesenteric cystic teratoma in children. Saudi Med J. (2003) 24(12):1388. 10.202207/article.14710290 14710290

[8] Okada T Sasaki F Onodera Y Oonishi S Ichikawa N Itoh T. Multiple mesenteric teratomas: usefulness of spiral computed tomography with 3-dimensional reconstruction. J Pediatr Surg. (2006) 41(4):868–71. 10.1016/j.jpedsurg.2005.12.040 16567213

[9] Rattan KN Ratan SK Jhanwar A Kaushik V Magu S. Immature mesenteric teratoma causing intestinal obstruction. Indian J Pediatr. (2007) 74(2):207–8. 10.1007/s12098-007-0020-3 17337839

[10] Singh M Ratan K Rani B Kadian Y Hasija S. Infected mature teratoma of mesentery in a child. APSP J Case Rep. (2013) 4(2):18 PMID: 24040596. 24040596 PMC3754403

[11] Zhang X Zhao Y Chen X Hua K. Mature mesenteric teratoma in a child: a case report. Clin Exp Obstet Gyn. (2015) 42(5):714. 10.12891/ceog1952.2015 26524836

[12] Li J Li S Xiao D Song J Mao J Yin J. Extratesticular gliomatosis peritonei after mesenteric teratoma: a case report and literature review. J Int Med Res. (2021) 49(9):030006052110470. 10.1177/03000605211047076 PMC8485300 34586943

[13] Ray R Dey S Khatun F Barman S Das M Chatterjee U. Adrenal and mesenteric teratomas in infants: common tumors in uncommon sites. J Indian Assoc Pediatr Surg. (2022) 27(3):354–6. 10.4103/jiaps.JIAPS_26_21 35733597 PMC9208687

[14] Kurtz RJ Heimann TM Beck AR Holt JA. Mesenteric and retroperitoneal cysts. Ann Surg. (1986) 203(1):109–12. 10.1097/00000658-198601000-00017 3942415 PMC1251046

[15] Ranganath SH Lee EY Eisenberg RL. Focal cystic abdominal masses in pediatric patients. Am J Roentgenol. (2012) 199(1):W1–16. 10.2214/AJR.11.6642 22733917

[16] Shah U Goldstein AM Gee MS Deshpande V. Case 24-2017. New Engl J Med. (2017) 377(6):574–82. 10.1056/NEJMcpc1616393 28792868

[17] Kim HHR Hull NC Lee EY Phillips GS. Pediatric abdominal masses. Radiol Clin N Am. (2022) 60(1):113–29. 10.1016/j.rcl.2021.08.008 34836559

